# Impact of cannabidiol on reward- and stress-related neurocognitive processes among individuals with opioid use disorder: A pilot, double-blind, placebo-controlled, randomized cross-over trial

**DOI:** 10.3389/fpsyt.2023.1155984

**Published:** 2023-03-30

**Authors:** Joji Suzuki, Sara Prostko, Veronica Szpak, Peter R. Chai, Primavera A. Spagnolo, Ruth E. Tenenbaum, Saeed Ahmed, Roger D. Weiss

**Affiliations:** ^1^Department of Psychiatry, Brigham and Women’s Hospital, Boston, MA, United States; ^2^Harvard Medical School, Boston, MA, United States; ^3^Department of Emergency Medicine, Brigham and Women’s Hospital, Boston, MA, United States; ^4^Department of Psychosocial Oncology and Palliative Care, Dana Farber Cancer Institute, Boston, MA, United States; ^5^The Koch Institute for Integrated Cancer Research, Massachusetts Institute of Technology, Cambridge, MA, United States; ^6^The Fenway Institute, Boston, MA, United States; ^7^Rutland Regional Medical Center, Rutland, VT, United States; ^8^Division of Alcohol, Drugs, and Addiction, McLean Hospital, Belmont, MA, United States

**Keywords:** opioid use disorder, cannabidiol, cue-induced craving, buprenorphine, methadone

## Abstract

**Introduction:**

Opioid use disorder (OUD) continues to be a significant public health concern. Medications for OUD (MOUD) such as buprenorphine reduce overdose mortality, but relapses occur often, leading to adverse outcomes. Preliminary data suggest that cannabidiol (CBD) may be a potential adjunctive treatment to MOUD by attenuating cue-reactivity. This pilot study sought to evaluate the impact of a single dose of CBD on reward- and stress-related neurocognitive processes implicated in relapse among those with OUD.

**Methods:**

The study was a pilot, double-blind, placebo-controlled, randomized cross-over trial aimed at assessing the effects of a single dose of CBD (Epidiolex®) 600 mg or matching placebo administered to participants with OUD receiving either buprenorphine or methadone. Vital signs, mood states, pain, opioid withdrawal, cue-induced craving, attentional bias, decision-making, delayed discount, distress tolerance, and stress-reactivity were examined at each testing session on two separate testing days at least 1 week apart.

**Results:**

Ten participants completed all study procedures. Receipt of CBD was associated with a significant decrease in cue-induced craving (0.2 vs. 1.3, *p* = 0.040), as well as reduced attentional bias toward drug-related cues as measured by the visual probe task (−80.4 vs. 100.3, *p* = 0.041). No differences were found among all the other outcomes examined.

**Discussion:**

CBD may have promise as an adjunct to MOUD treatment by attenuating the brain response to drug-related cues, which, in turn, may reduce the risk of relapse and overdoses. Further research is warranted to evaluate the potential for CBD as an adjunctive therapy for individuals in treatment for OUD.

**Clinical Trial Registration:**

https://clinicaltrials.gov/ct2/show/NCT04982029.

## Introduction

Fueled by the emergence of illicit fentanyl, an unprecedented epidemic of opioid overdoses among individuals with opioid use disorder (OUD) continues to unfold in the United States (1). In 2021, there were over 100,000 overdose deaths, the most ever recorded in a 1-year period in the United States (2). Medications for OUD (MOUD), such as buprenorphine or methadone, are critically important tools to address this crisis by suppressing illicit opioid use, increasing retention in OUD treatment, and reducing overdose mortality by up to 70% (3–6). Unfortunately, many patients discontinue MOUD treatment too early, partly in response to the emergence of craving after exposure to drug-related cues and stressors, resulting in relapse (7–9). Psychosocial interventions, such as cognitive-behavioral therapy, have excellent empirical support for substance use disorders, but have not been as helpful in improving OUD-related outcomes (10). Therefore, we urgently need innovative interventions that help prevent patients on MOUD from relapsing.

Cannabidiol (CBD), a non-addictive and non-psychoactive constituent of the cannabis plant has received increasing scientific attention as a possible treatment for psychiatric and substance use disorders (11–13). While the mechanism of action remains largely speculative, CBD manifests negative allosteric properties at CB1, shows only a low degree of affinity for CB1 and CB2 receptors, and exerts a broad range of pharmacologic actions through other receptors (14, 15). In both pre-clinical and human studies, CBD appears to reduce attentional bias to drug-related cues, cue-induced craving, and cue-induced drug reinstatement (16–21). However, no prior studies have evaluated the impact of CBD on a broader range of reward- and stress-related neurocognitive processes implicated in opioid relapse.

Accordingly, the aim of this pilot study was to evaluate the impact of CBD on reward- and stress-related neurocognitive processes among individuals with OUD maintained on buprenorphine or methadone. This line of research, if successful, could help pave the way for utilizing CBD as an adjunct to MOUD in reducing the risk of relapse and preventing overdoses.

## Methods

### Overview

The study was a pilot, double-blind, placebo-controlled, cross-over trial of a single dose of CBD 600 mg or matching placebo administered on two separate testing days at least 1 week apart, conducted in a controlled laboratory setting. The study was approved by the Mass General Brigham (MGB) Human Research Committee, Institutional Review Board for Rutland Regional Medical Center (RRMC), and registered with ClinicalTrials.gov (NCT04982029). Prior to study initiation, an investigational new drug exemption from the Food and Drug Administration was obtained for administration of CBD.

### Setting

The study was conducted at the outpatient research facilities of the Center of Clinical Investigation at Brigham and Women’s Hospital (BWH) in Boston, MA, and on site at RRMC, Rutland, VT. Recruitment and data collection occurred between September 2021 and December 2022.

### Study participants

Potential participants were recruited *via* flyers and referral from clinical programs at BWH and RRMC, as well as from using online advertisements. Inclusion criteria were adults 18 years or older, having a DSM-5 diagnosis of OUD, receiving treatment with buprenorphine or methadone, and agreeing to abstain from cannabis or CBD products for the duration of the trial. Exclusion criteria were the self-reported use of any CBD-containing products in the past 30 days, the need for any inpatient level treatment for substance use or psychiatric disorders, history of any psychotic disorder, currently pregnant, hepatic enzymes greater than three times the upper normal limit, hypersensitivity to cannabinoids or sesame oil, and currently taking any medications with known significant pharmacokinetic interactions with CBD.

### Overall study procedures

Potential participants were screened over the telephone to determine preliminary eligibility. Those meeting preliminary eligibility were invited for a baseline visit, during which written informed consent was obtained. Subsequently, baseline assessments and laboratory tests were conducted. Those meeting the full inclusion and exclusion criteria were then scheduled for two additional study visits, at least 1 week apart. During each study visit, participants received either a single dose of CBD 600 mg or matching placebo in double-blind fashion, with the order randomized and counterbalanced. The investigational drug service at BWH or the pharmacy at RRMC created the randomization code and prepared the CBD and the matching placebo.

### CBD

CBD (GW Pharmaceuticals Epidiolex®) was purchased as a 100 mg/mL oral solution.

### Placebo

Matching placebos were created using sesame oil and strawberry flavoring, and drawn into oral syringes.

### Baseline visit

After obtaining written informed consent, the following assessments were completed: Time Line Follow-Back (TLFB) (22), Patient Health Questionnaire (PHQ9) (23), Generalized Anxiety Disorder-7 (GAD7) (24) Brief Pain Inventory (BPI) (25), Positive and Negative Affect Scale (PANAS) (26), and Clinical Opioid Withdrawal Scale (COWS) (27). Vitals signs, liver function tests, urine immunoassay screen for common drugs of misuse, and pregnancy test (if female) were obtained.

### Study visits

Participants were scheduled for two study visits, at least 1 week apart. After arrival, participants received CBD or placebo, and waited 1 h before completed the assessments except for baseline vital signs, which were obtained immediately after receipt of CBD or placebo. If the participant received CBD during the first visit, they received placebo during the second visit, and vice versa. Double-blind was maintained throughout the trial.

#### Vitals signs

Heart rate and blood pressure measurements were obtained immediately following administration of CBD or placebo, and repeated every 30-min post-dose.

#### Cue-induced craving paradigm

Participants were asked to report their craving for opioids (pre-cue) prior to the cue-reactivity paradigm on a visual analog scale of 0 to 10 (28, 29). The study utilized a total of 40 drug-related and 10 neutral images shown on computer screen using a standardized protocol used in previous studies (29). Drug images were similarly matched to the neutral images in composition and style, and utilized images that have evoked strong responses in prior studies (19, 28). Participants viewed, in random order, either the drug-related images or the neutral images for a total of 1 min. Following the stimuli presentation, participants rated their craving (post drug-cue or post neutral-cue) on a visual analog scale of 0 to 10. Following that, participants repeated the procedure with the drug-related images or the neutral images, whichever they had not yet observed during that study visit. The order in which the drug-related or neutral cues were presented was randomized and counterbalanced. The cue exposure procedure ended with a standardized relaxation and debriefing exercise. To limit habituation from repeated exposure, out of the 40 drug-related images, 20 were using during one visit, and the other 20 during the other visits.

#### Visual probe task

A total of 20 illicit opioid-related images and 8 composition-matched neutral images were utilized. Each trial began with a fixation point (500 ms), and a pair of images were then shown on the computer screen for either a short (200 ms) or long (500 ms) duration (18, 30). The former assessed automatic orienting, while the latter assessed controlled attention processing. Images were either a pair of drug-related and neutral images, or just neutral images. The location and order of the images were randomized and counterbalanced. After presentation, the images were replaced with a single probe, behind either the left or right image. The probe remained visible until the participant responded to identify the location of the probe by pressing the response keys as quickly as possible. The trial was set up to repeat a pair of images if the participant chose incorrectly. Each image pair was presented a total of eight times, producing 80 critical trials and 32 neutral trials. The task was programmed using ePrime 3.0 software, and the reaction time was measured using the Chronos device (Psychology Software Tools, Pittsburgh, PA).

#### Monetary choice questionnaire

The Monetary choice questionnaire (MCQ) is a self-administered questionnaire with 27 items, in which participants choose between a smaller, immediate reward and a larger, delayed reward (e.g., “Would you prefer $55 today, or $75 in 61 days?”) (31). The MCQ is scored by calculating by identifying the point at which the respondent demonstrates indifference between the immediate and delayed reward using a reference discounting curves.

#### Iowa gambling task

The computerized Iowa gambling task (IGT) is a game in which players are presented with four decks of cards and an endowment of fake money (e.g., $2,000), and are instructed to maximize profits over 100 cycles by selecting cards from any deck (32). Each selected card results in a reward or a penalty. The goal of this task is to lose as little money as possible, while earning as much as possible. Two of the decks (A and B) contain larger rewards (e.g., $100) but also larger penalties, while the other two decks (C and D) contain smaller rewards (e.g., $50) but also smaller penalties. Playing from decks A and B leads to greater losses, while playing from decks C and D will maximize overall gain. The task was administered using ePrime 3.0 software.

#### Mirror tracing persistence task

Mirror tracing persistence task (MPTP) is a computerized stress-induction paradigm in which participants trace a shape on the computer using the mouse, but to make the task distressing, the cursor moves in the opposite direction of the mouse (33, 34). Whenever an error is made or the participant takes too long to move the cursor, the cursor is returned to the starting position, and a loud aversive sound is played. The cursor would also beep and return to the starting position if the participant took too long to move the cursor. Each task was 2 min in duration, repeated three times, with the third attempt serving as a measure of distress tolerance. This task has been used in prior studies successfully with individuals with OUD (34).

#### Salivary cortisol

As a measure physiologic stress-reactivity as induced by the MTPT, salivary cortisol was measured by collecting saliva, using standard collection tubes at baseline, immediately after and 20 min after the MTPT.

#### PANAS

In addition to physiologic reactivity, subjective stress-reactivity was measured using the negative affect subscale of the PANAS, measured at baseline, immediately after, and 20 min after the MTPT.

### Analytic strategy

Descriptive statistics were used to summarize the data. The differences in heart rate, systolic blood pressure, and diastolic blood pressure recordings at 60 min post-dosing of CBD or placebo and at baseline values were compared. Cue-induced craving was calculated as the difference in post-cue craving scores in response to drug-related cues minus pre-cue craving scores. Attentional bias was calculated as the difference in reaction time to neutral cues and drug-related cues among drug-neutral trials with a correct response, such that positive scores indicated attentional bias toward drug-related cues. Scores from the IGT were calculated as the sum of the numbers selected from decks C and D, minus the number of cards selected from decks A and B. Delayed discounting was calculated using a standardized conversion from the MCQ. Distress tolerance was measured as the number of seconds that participants persisted in the third trial of the MPTP. Paired t-test was used in all analyses, with alpha set at 0.05.

## Results

**Figure 1:**
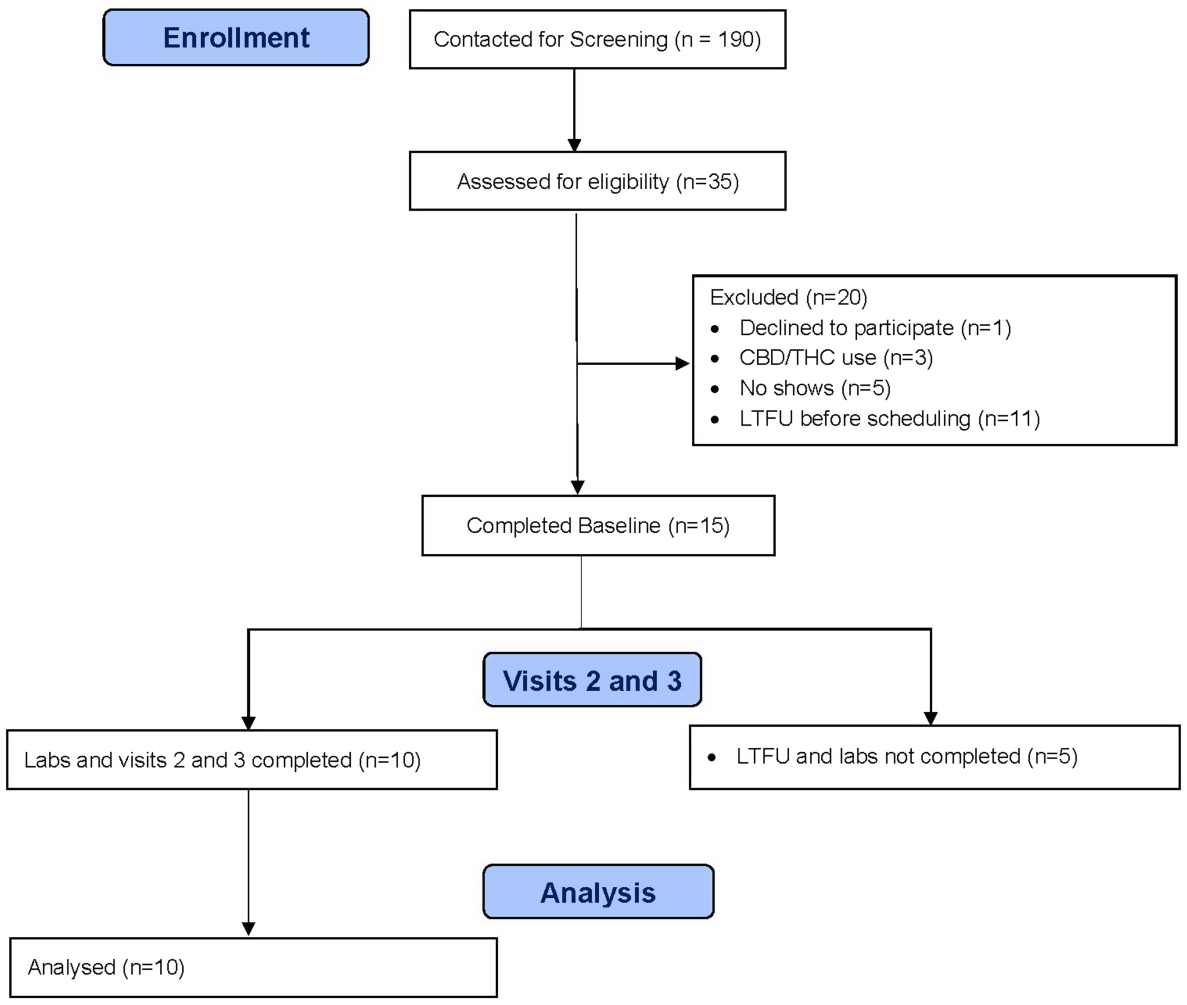
Study CONSORT flow diagram.

A total of 10 participants completed all study procedures (see Figure 1). Participant characteristics are summarized in Table 1. Overall, they averaged 45.1 years old (SD 9.1), 50% were female, all were white, and one was Hispanic. Psychiatric comorbidities were common, including major depressive disorder (70%), generalized anxiety disorder (70%), post-traumatic stress disorder (70%), and attention deficit hyperactivity disorder (50%). All participants had an OUD, but other substance use disorders were common, including cocaine (30%), alcohol (30%), tobacco (30%), and cannabis (10%). Participants were equally split between receiving buprenorphine treatment and methadone maintenance. Consistent with prior studies, CBD was well-tolerated in our sample, with no reported adverse events. While none of the participants self-reported any CBD use in the 30 days prior to the trial nor during the trial itself, 3 participants tested positive for THC and 1 participant tested positive for cocaine during the trial.

**Table 1:** Summary of participant characteristics.

	Total *n* = 10
Age (years), *n* (SD)	45.1 (9.1)
Sex (F), *n* (%)	5 (50%)
Race (White), *n* (%)	10 (100%)
**Ethnicity, *n* (%)**	
Non-Hispanic	9 (90%)
Hispanic	1 (10%)
**Marital status, *n* (%)**	
Married	1 (10%)
Separated/Divorced	4 (40%)
Single	5 (50%)
**Employment, *n* (%)**	
Full-time	2 (20%)
Unemployed	8 (80%)
**Psychiatric history, *n* (%)**	
Major depressive disorder	7 (70%)
Generalized anxiety disorder	7 (70%)
Panic disorder	1 (10%)
Post-traumatic stress disorder	7 (70%)
Bipolar disorder	1 (10%)
Attention deficit hyperactivity disorder	5 (50%)
**Substance use disorder history, *n* (%)**	
Opioid use disorder	10 (100%)
Cocaine use disorder	3 (30%)
Amphetamine use disorder	0
Alcohol use disorder	3 (30%)
Sedative-hypnotic use disorder	0
Cannabis use disorder	1 (10%)
Tobacco use disorder	3 (30%)
**Medications for opioid use disorder, *n* (%)**	
Buprenorphine	5 (50%)
Methadone	5 (50%)

Results of the assessments are summarized in Table 2. Participants generally reported increased craving in response to exposure to drug-related stimuli as compared to baseline, with the intensity of the craving significantly lower for those in the CBD arm (0.9 vs. 2.4, *p* = 0.0046). Cue-induced craving was also significantly lower in the CBD arm (0.2 vs. 1.3, *p* = 0.040). In addition, attentional bias to drug-related cues for the automatic orienting (200 ms condition) was significantly lower in the CBD arm, indicating greater bias toward the neutral images (−80.4 ms vs. 100.3 ms, *p* = 0.041) but not for the controlled attention (500 ms condition, 145.2 ms vs. −93.1 ms *p* = 0.28). Changes in vital signs, pre-cue craving, PHQ-9, GAD-7, BPI severity, BPI interference, PANAS positive affect, PANAS negative affect, COWS scores, IGT, MCQ, MTPT, and stress-reactivity were not significantly different between the CBD and placebo arms.

**Table 2:** Summary of reward- and stress-related neurocognitive findings (*n* = 10).

	CBD 600 mg	Placebo	*p*
**Vitals (baseline to 60 min)**			
Heart rate	−9.3 (SD 7.9)	−5.3 (SD 6.2)	0.15
SBP	−11.1 (SD 6.4)	−13.6 (SD 10.8)	0.50
DBP	−5.0 (SD 7.6)	−3.0 (SD 9.1)	0.53
PHQ (range 0–27)	8.9 (SD 6.6)	8.0 (SD 5.5)	0.34
GAD7 (range 0–21)	8.1 (SD 6.1)	8.6 (SD7.4)	0.72
**BPI (range 0–10)**			
Severity	4.2 (SD 2.3)	3.3 (SD 2.4)	0.31
Interference	4.0 (SD 3.1)	3.3 (SD 2.1)	0.40
**PANAS (range 0–50)**			
Positive	28.4 (SD 8.4)	31.6 (SD 7.4)	0.19
Negative	18.0 (SD 9.9)	21.2 (SD 11.2)	0.24
**COWS (range 0–48)**	1.3 (SD 1.6)	1 (SD 1.8)	0.65
**Craving (range 0–10)**			
Pre-cue	0.7 (SD 1.3)	(SD 2.0)	0.37
Post drug-cue	0.9 (SD 1.1)	2.4 (SD 1.7)	0.0046*
Post neutral-cue	0.5 (SD 1.3)	1.1 (SD 2.0)	0.081
Cue-induced craving	0.2 (SD 0.79)	1.3 (SD 1.9)	0.040*
**Attentional bias**			
Automatic orienting	−80.4 (SD 298.8)	100.3 (SD 123.5)	0.041*
Controlled attention	145.2 (SD 392.3)	−93.1 (SD 268.5)	0.28
**Iowa gambling task**	2.2 (SD 9.3)	−6.4 (SD 18.9)	0.074
**Monetary choice questionnaire**	0.020 (SD 0.024)	0.039 (SD 0.075)	0.35
**Mirror tracing persistence task**	59.0 (SD 37.7)	99.8 (SD 100.8)	0.28
**Stress-reactivity**			
Salivary cortisol	−0.016 (SD 0.052)	−0.16 (SD 0.23)	0.14
Negative mood	3.6 (SD 6.9)	0.89 (SD 2.4)	0.26

*p<0.05.

## Discussion

Although MOUDs are critically important evidence-based tools in reducing overdose mortality and increasing treatment retention, innovative pharmacologic and behavioral approaches to further improve clinical outcomes are urgently needed (6). The study reported here is the first randomized trial to our knowledge to examine the effects of a single dose of CBD on reward- and stress-related neurocognitive processes among individuals with OUD who were actively being treated with MOUDs. In contrast, prior trials of CBD for OUD enrolled individuals who were not receiving any MOUDs (20, 21). Consistent with prior studies, results showed that a single dose of CBD significantly reduced cue-induced craving among individuals with OUD (19, 21). Targeting cue-reactivity has scientific merit, given that individuals with OUD with greater subjective cravings in response to cues are significantly more likely to return to opioid use at an earlier time point (35). Indeed, subjective drug craving is a well-described precipitant for subsequent substance use in opioid as well as other substance use disorders (36–40). Nevertheless, the overall increase in craving in response to drug-related cues was mild in this particular trial, making it difficult to know to what extent this attenuation will have any impact on the risk for relapse and overdose among individuals in MOUD treatment.

As a novel finding, our results indicated that a single dose of CBD, compared to placebo, led to a significant reduction in attentional bias to drug-related cues under the automatic orienting condition but not under the controlled attention condition. When the image is displayed only for 200 ms under the automatic orienting condition, the human eye is incapable of shifting the gaze from one image to the other (30, 41). If there is attentional bias toward drug-related cues, as hypothesized in the incentive salience model of addiction, the reaction time would be smaller than that to neutral cues, resulting in a negative value (42). Therefore, the results are consistent with the growing evidence base that CBD attenuates the reward circuitry’s response to salient drug cues (13). This finding is similar to that reported by Hindocha and colleagues, in which a single dose of CBD 800 mg, compared to placebo, significantly reduced attentional bias to tobacco-related cues among cigarette smokers (18). Similarly, in a study of individuals who smoke cannabis regularly, those smoking strains with high CBD content displayed reduced attentional bias to cannabis-related images (17). Taken together, these results therefore suggest not only that reactivity to cues may persist even while on MOUDs, but also that CBD may serve as an adjunctive treatment to MOUDs by attenuating attentional bias and cue-reactivity. Additionally, this work may suggest that CBD could be beneficial for individuals treated by MOUDs who have persistently high cue-reactivity as a strategy to reduce relapse risk and encourage adherence.

Contrary to prior studies, our results did not demonstrate any significant reduction in stress-reactivity after a single dose of CBD (43–45). The null finding may be due to the relatively low intensity of the stress-induction paradigm employed by this trial, although a prior trial has indicated this should have been sufficient to elicit a response (34). Nevertheless, it remains unclear to what extent attenuation of stress-reactivity helps to reduce the risk relapse in those with OUD, given the mixed findings to date regarding the association between stress-reactivity and craving as well as the risk of relapse (35, 43, 46). Our results also indicated that a single dose of CBD did not significantly (*p* = 0.074) improve impulsive decision-making as assessed using the Iowa Gambling Task. Individuals with substance use disorders generally demonstrate risky decision-making, where decisions are made impulsively (47). However, the negative result warrants confirmation with further research given that this study may not have been powered sufficiently. Results from this study also failed to show that a single dose of CBD improved delayed discount or distress tolerance, both known to be impaired among individuals with substance use disorders (31, 48–50). Given that deficits in decision-making, delayed discounting, and distress tolerance contribute to the risk of relapse, further research to assess CBD’s impact on these processes using larger sample size and multiple dosing may be warranted.

There are several limitations to the study. Despite the use of a double-blind randomized cross-over design, this was a small pilot study, necessitating larger studies to replicate and confirm the study findings. While we followed standard procedures for assessing cue-reactivity, report of craving remains subjective and is susceptible to bias. We excluded individuals reporting any CBD use in the 30 days prior to the trial, but we did not confirm this biochemically through toxicology testing. Despite asking participants to abstain from cannabis use during the trial, 2 participants had a positive test at baseline, and 3 tested positive during the trial, potentially introducing confounders. While information regarding concurrent psychiatric medications were collected at screening, we did not systematically record nor confirm the dosage or other information regarding any concurrent psychiatric medications, potentially introducing additional confounders.

In summary, CBD was a well-tolerated pharmacologic intervention that has a potential to be an adjunctive treatment to MOUD to reduce the risk of relapse by attenuating cue-reactivity. Additional research on the effects of CBD is warranted to better understand the potential role of CBD in improving clinical outcomes of individuals with OUD treated with MOUD.

## Data availability statement

The raw data supporting the conclusions of this article will be made available by the authors, without undue reservation.

## Ethics statement

The studies involving human participants were reviewed and approved by Mass General Brigham Institutional Review Board. The patients/participants provided their written informed consent to participate in this study.

## Author contributions

JS: design and data analysis. JS, SP, VS, PC, RT, and SA: data collection. JS, SP, VS, PC, PS, SA, and RW: interpretation of the data. JS, SP, VS, PC, PS, RT, SA, and RW: writing and editing of the manuscript. All authors contributed to the article and approved the submitted version.

## Funding

This work was supported by the Harvard Medical School Broderick Phytocannabinoid Research Initiative Faculty Research Grant (JS), and in part by the National Institutes of Health [K23DA042326 (JS), K23DA044874 (PC) and R44DA051106 (PC)], Hans and Mavis Lopater Psychosocial Foundation (PC), and Defense Advanced Research Projects Agency HR001120S0041 (PC).

## Conflict of interest

RW has consulted to Alkermes.

The remaining authors declare that the research was conducted in the absence of any commercial or financial relationships that could be construed as a potential conflict of interest.

## Publisher’s note

All claims expressed in this article are solely those of the authors and do not necessarily represent those of their affiliated organizations, or those of the publisher, the editors and the reviewers. Any product that may be evaluated in this article, or claim that may be made by its manufacturer, is not guaranteed or endorsed by the publisher.
